# Acupuncture for Adolescent Depression Disorder: protocol for a randomized controlled trial

**DOI:** 10.3389/fpsyt.2025.1597093

**Published:** 2025-07-14

**Authors:** Yuting Duan, Yuejuan Cai, Junting Lai, Mengxi Zhang, Zewei Chen, Haichun Yang, Shujuan Liu, Yuening Deng, Enyi Liu, Feng Jiang, Zhirui Xu, Weifeng Zhu, Lin Yu

**Affiliations:** ^1^ The Affiliated Guangzhou Hospital of TCM of Guangzhou University of Chinese Medicine, Guangzhou, China; ^2^ The Affiliated Traditional Chinese Medicine Hospital, Guangzhou Medical University, Guangzhou, China

**Keywords:** acupuncture, Adolescent Depression Disorder, RCT, protocol, clinical

## Abstract

**Background:**

Adolescent Depression Disorder (ADD) is a disease with a high rate of disability and death worldwide, and its incidence is increasing, seriously influencing the physical and mental health of adolescents. Acupuncture is a complementary alternative therapy that has achieved good clinical efficacy in the intervention of depression, but its efficacy in ADD is uncertain. Therefore, the aim of this pilot trial is to preliminarily explore the possibility of acupuncture in the treatment of ADD, and to evaluate the feasibility of conducting further large-scale clinical trials to verify the efficacy of acupuncture.

**Methods:**

In this pilot randomized controlled trial, 60 participants will be randomly assigned to receive acupuncture or sham acupuncture for 8 weeks of treatment and 4 weeks of follow-up. The primary outcome will be the difference in Children’s Depression Rating Scale-revised (CDRS-R) scores at weeks 0 and 8 of treatment. Secondary outcomes will include 17-item Hamilton Depression Scale (HAMD-17) clinical score reduction rate and clinical remission rate, difference in CDRS-R scores, HAMD-17 scores, 14-item Hamilton Depression Scale (HAMD-14) scores, Pittsburgh Sleep Quality Index scale (PSQI) scores, and Columbia-Suicide Severity Rating Scale (C-SSRS) scores. All outcomes will be assessed at weeks 0, 2, 4, 6, 8 of treatment and week 12 of the follow-up period. In addition, we will assess the safety outcomes from baseline to the end of this trial and feasibility outcome after treatment.

**Ethics and dissemination:**

The trial protocol is in accordance with the principles of the Declaration of Helsinki, and has been approved by the Ethics Committee of the Affiliated Traditional Chinese Medicine Hospital, Guangzhou Medical University (approval number 2024NK73). The results of this trial will be made publicly available on the registration platform. Professional writers will not be used for this trial. Future authorship of trial publications will be based on the authors’ contributions.

**Discussion:**

The results of this study will provide information on the efficacy and safety of acupuncture in the treatment of add, and evaluate the feasibility of acupuncture in the treatment of add, which will provide the basis for further clinical intervention and scientific research.

**Clinical Trial Registration:**

http://itmctr.ccebtcm.org.cn/zh-CN/UserPlatform/ProjectView?pid=730cb4d2-3634-4789-a51d-85a20514aa0a, identifier ITMCTR2024000186.

## Background

Depression is a common mental disorder characterized by loss of interest or pleasure, sadness, lack of self-worth, and suicidal tendency (1). Globally, an estimated 5.0% of adults suffer from depression (2), and more than 350 million people are affected by depression (3). Adolescence is a unique period of growth, a stage of sudden physiological, biochemical, endocrine and psychological and behavioral changes, all of which can make adolescents vulnerable to mental health problems. In China, more than 95 million people suffer from depression, with 30.28% of young people under the age of 18 (4). Globally, one in seven adolescents aged 10–19 suffers from a mental disorder, accounting for 13% of the global burden of disease in that age group, and depression is one of the leading causes of illness and disability among adolescents (5). The rise in the incidence of depression among adolescents is particularly worrying since depression is also closely related to suicide, and the suicide rate among adolescents has been on the rise for more than a decade (6). Therefore, ADD have become one of the major public health issues worldwide (7).

Guidelines generally recommend active support and monitoring or psychotherapy for patients with mild depressive disorders, and selective serotonin reuptake inhibitor (SSRI) medications or a combination of psychotherapy and SSRIs for patients with moderate or severe disorders and for patients with mild disorders who do not improve (8). As a recommended treatment, the efficacy of Psychotherapy in ADD is not entirely satisfactory. In a meta-analysis of 366 randomized clinical trials including 36072 patients comparing psychotherapy with control conditions, psychotherapies had lower effect sizes in children and adolescents compared with adults (9). In addition to this, there are many challenges to the widespread use of psychotherapy, including high costs, limited availability of therapists, and insufficient access to treatment. Currently, SSRIs is the recommended first-line pharmacological treatment for ADD (10). However, the choice of SSRIs is also limited. For example, the Food and Drug Administration (FDA) approves only fluoxetine and escitalopram for the treatment of depression in adolescents, but other SSRIs and serotonin–norepinephrine reuptake inhibitors (SNRIs), such as venlafaxine, are commonly used off label for this purpose (6). Long-term use of SSRIs to treat depressive disorders in adolescents may lead to a variety of adverse reactions, including insomnia, nausea or headache, tremors, dry mouth (11), and may even increase the risk of suicidal thoughts and behaviors in adolescents (12). In addition, about 40% of them fail the treatment either with antidepressant medication or evidence‐based psychotherapy (13). Therefore, there is an urgent clinical need for a new treatment for ADD with good efficacy and low side effects.

Acupuncture is currently one of the most popular complementary alternative therapies internationally (14). Accumulating evidence from clinical studies suggests that acupuncture can play a vital role in the treatment of depressive symptoms, with significant reductions in depressive symptoms compared to normal care or psychotherapy alone (15–17), and it is safer and better tolerated than antidepressants alone, with better improvements in quality of life (18, 19). A Meta-analysis (20) showed that acupuncture reduced HAMD scores in adult patients with depressive disorders compared to comfort acupuncture. For ADD, Guideline for adolescent depression in prevention and treatment with integrated Chinese and western medicine suggest that acupuncture may be used for treatment (21). And a study on adolescent depressed rats proved that taVNS is capable of ameliorating adolescent depressive and anxiety like behaviors by regulating plenty of genes in the three brain regions (13). However, there is no clear clinical evidence that acupuncture is effective in ADD, so we designed this pilot trial to preliminarily explore the possibility of acupuncture in the treatment of ADD, and to evaluate the feasibility of conducting further large-scale clinical trials to verify the efficacy of acupuncture.

## Study design and methods

### Study design

This is a single-center randomized controlled trial, and participants will be assigned to receive acupuncture or sham acupuncture in a 1:1 ratio. Each participant will sign an informed consent form prior to enrollment. The protocol followed SPIRIT for TCM statement (22).

**Figure 1:**
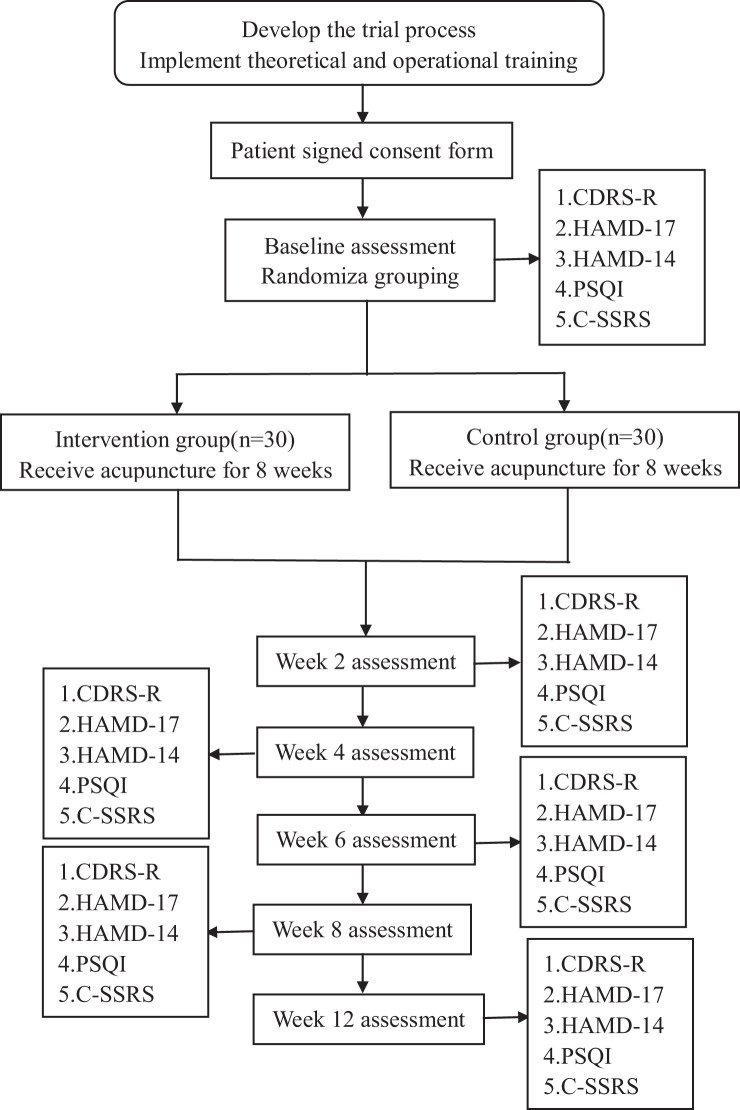
Flowchart of study process.

The total observation period will be 12 weeks, including an 8-week intervention period (weeks 0-8), and a 4-week follow-up period (weeks 8-12). Assessments will be conducted at baseline, week 2, week 4, week 6, week 8 and week 12. The study process is illustrated in **Figure 1**, while the schedule of enrolment, treatments, and assessments can be seen in **Table 1**.

**Table 1:** .

Time (week)	Baseline	Treatment period				Follow-up
content	0	2	4	6	8	12
Patient Consent Form	✓					
Enrollment Screening Form	✓					
Demographic information	✓					
History of present illness	✓					
Family history	✓					
Past history	✓					
Personal history	✓					
CDRS-R	✓	✓	✓	✓	✓	✓
HAMD-17	✓	✓	✓	✓	✓	✓
HAMA-14	✓	✓	✓	✓	✓	✓
PSQI	✓	✓	✓	✓	✓	✓
C-SSRS	✓	✓	✓	✓	✓	✓
Safety evaluation	✓				✓	
Random form	✓					
Drug combination	✓					
Adverse Event record form	✓					
Serious Adverse Event record form	✓					
Table of trial completion and early termination	✓					
CRF Audit Statement	✓					

### Participants

The researchers will select 60 patients diagnosed with ADD by at least two specialists between October 2024 and July 2025 at the Department of Sleep Psychology, Acupuncture and Encephalopathy of the Affiliated Traditional Chinese Medicine Hospital, Guangzhou Medical University.

### Diagnostic criteria

In the DSM-5, ADD is classified under “depressive disorders”. Therefore, this study will use the DSM-5 diagnostic criteria for depressive disorders as the diagnostic criteria.

### Inclusion criteria

Participants who meet the following inclusion criteria will be included: (1) 12 years ≤ age <18 years; (2) Meet DSM-5 diagnostic criteria for “depressive disorders”; (3) Meet mild to moderate ADD (8≤HAMD-17 score < 20); (4) No suicide risk, “No” to all items on the C-SSRS, and a score <3 on item 3 (suicide) of the HAMD-17; (5) No medication (escitalopram, mirtazapine, etc.) or physiotherapy (transcranial magnetic stimulation, transcranial electrical stimulation, acupuncture, electroconvulsive therapy, etc.) received within the past 1 month; (6) Refuse to take the medication; (7) Good reading and writing ability, and able to communicate normally; (8) Volunteer to participate in this study and sign an informed consent form.

### Exclusion criteria

Participants who meet any of the following criteria will be excluded: (1) Meet the DSM-5 diagnosis for other mental disorders such as bipolar disorder, schizophrenia, post-traumatic stress disorder, and personality disorders currently or previously; (2) With severe or unstable cardiovascular, respiratory, hepatic, renal, hematological or other systemic diseases, as well as acute illnesses, infectious diseases, malignant tumors; (3) Psychoactive substance (e.g., alcohol, drugs, etc.) abuse or dependence history.

### Dropout criteria

Participants who meet any of the following criteria will be considered as dropped out: (1) Self-exit after joining the trial; (2) Lost contact in the middle of the trial for various reasons; (3) Write incomplete or inaccurate clinical information that may affect the results of the trial.

### Removal criteria

Participants who meet any of the following criteria will be removed: (1) Receive a treatment prohibited for this trial; (2) Poor compliance, not completing case observation forms, examinations or treatment as required after inclusion.

### Discontinuation criteria

The trial leader will obtain these interim results and make the final decision to terminate the trial if the following occurs: (1) Participants suffer a severe illness or adverse reaction during the trial; (2) Participants request to discontinue the trial; (3) Participants suffer self-injury, suicidal behavior, or have a change in diagnosis and treatment due to medical condition changes during the trial; (4) Contrary to the purpose and protocol of the study.

### Randomization and blinding

In this study, participants will be divided into an intervention group and a control group using a block randomization method. The randomization sequence will be generated by a randomizer not involved in trial implementation and statistics with the aid of Stata software, setting a block length of 4, with the 60 participants divided into a total of 10 blocks.

The random assignment sequence and grouping results of the Stata software output will be recorded by the randomizer on paper and sealed in an opaque envelope with a number representing the order in which the participants are grouped. Envelopes will be kept by randomizer.

After the participants meet the enrollment criteria, sign the informed consent and complete the baseline data collection, the researchers will ask for a random number according to the enrollment order of the participants from randomizer, and then conduct clinical studies according to the predetermined treatment plan of each group.

Participating researchers include randomizers, therapists, assessors and statistical analysts. The study will be blinded to patients, assessors and statistical analysts. If a participant experiences a serious adverse reaction during the trial, unblinding will take place immediately. The procedure for unblinding will be the intervener explains to the trial leader the occurrence of a serious adverse reaction in the participant and requests unblinding. Data from this study will be reviewed and evaluated by the data monitoring committee (DMC), which consisted of 5–6 clinicians and statisticians independent of the sponsor and competing interests. And every three months there will be a trial monitoring by an independent clinical trial monitor.

### Interventions

In this trial, the needles used in intervention group will be the Tianxie medical stainless steel sterile millimeters needle device (Suzhou Tianxie Medical Equipment Co., Ltd.), which consisted of two parts, the acupuncture needle and the foam pad. Acupuncture needles have a thickness of 0.3 mm and a specification of 1 inch (1inch=25mm). In control group will be the Tianxie medical special flat-tip needle device (Suzhou Tianxie Medical Equipment Co., Ltd.), which consisted of two parts, the flat-tip needle and the foam pad. Flat-tip needles have the same thickness and specification of acupuncture needles.

The acupoints in both the intervention and control groups selected will be GV20, GV29, HT7 (bilateral), CV17, PC6 (bilateral), LR3(bilateral), SP6(bilateral). The localization of the acupoints refer to the national standard of the People’s Republic of China, “Names and Localization of Acupoints” (GB/T12346-2021). The operation methods and precautions are standardized in accordance with the “Acupuncture and Moxibustion Technical Practice Guidelines-Needling” (GB/T21709.21).

The interventions in both groups will be conducted by an acupuncturist registered as a licensed TCM practitioner with at least 3 years of clinical experience in acupuncture practice. The acupuncturist will receive training on study protocol and standard acupuncture manipulation before study initiation.

Each participant will have their treatment sheet, which will record the date they are supposed to come for treatment, whether they receive the treatment on time or not, and will be signed by both the intervener and the participant, in an attempt to improve the participants’ compliance.

### Intervention group

The patient will be asked to take a sitting position. After the acupoints are localized, the operator’s hands and acupoints will be routinely sterilized with 75% alcohol, and the acupuncture needles will be inserted into the body by piercing the foam pads, with the specific depths of the needles as follows: ①GV20: 0.5 inches of flat stabbing; ②GV29: 0.3 inches of flat stabbing; ③HT7 (bilateral): 0.3 inches of straight stabbing; ④CV17: 0.3 inches of flat stabbing; ⑤PC6 (bilateral): 0.5 inches of straight stabbing; ⑥LR3 (bilateral): 0.5 inches of straight stabbing; ⑦SP6 (bilateral): 0.8 inches of straight stabbing. All acupoints will be selected with 1-inch acupuncture needles for needling, giving small and even lifting-thrusting and twirling after needle insertion to achieve the effect of “De qi”, which will be defined as local soreness and distension. After that, the needles will be left in place for 20 minutes. Frequency is 2 times a week for 8 weeks of treatment.

### Control group

**Figure 2:**
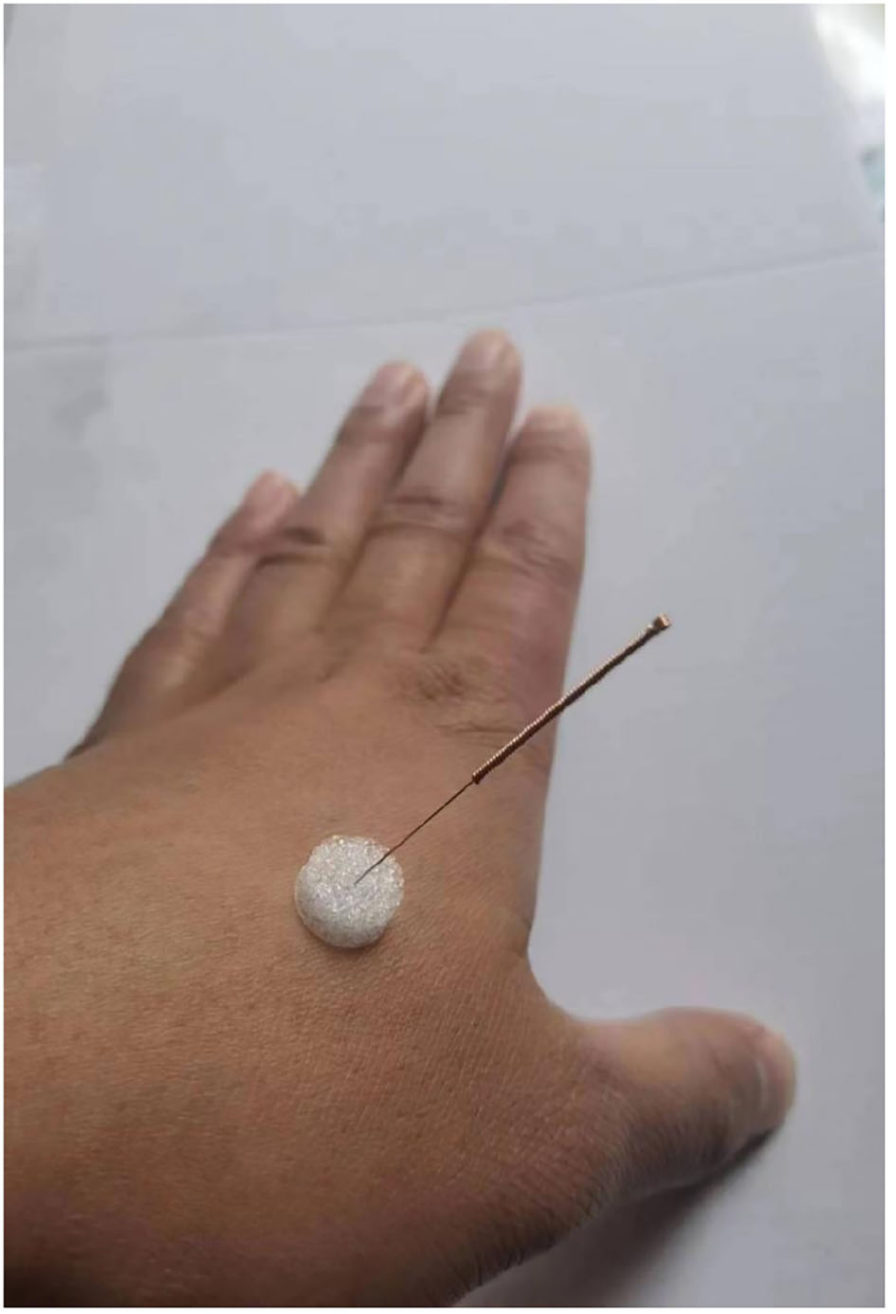
Schematic diagram of sham acupuncture.

The patient will be asked to take a sitting position. After the acupoints are localized, the operator’s hands and acupoints will be routinely sterilized with 75% alcohol, and select 1-inch special flat-tip needles (see **Figure 2** for details) to pierce the foam pads, with the tip of the needle against the skin of the acupoint through the base of the needling device. Giving small and even lifting-thrusting and twirling after needle insertion and the needles will be left in place for 20 minutes like the intervention group. Frequency is 2 times a week for 8 weeks of treatment.

### Observational indicators and follow-up

Baseline demographic data (age, gender, body mass index, smoking history, alcohol consumption history, place of residence, years of education, whether the only child, parents’ marital status, family’s area of residence, etc.) and disease information (age of onset, duration of disease, family history of psychiatric disorders, medication, etc.) will be collected from the participants. Participants will be followed up at 4 weeks after they have finished treatment (i.e., Week 12), either face-to-face or by telephone, to record changes in their medical condition and follow-up information. As well, they will be evaluated by CDRS-R, HAMD-17, HAMA-14, PSQI, and C-SSRS (see *Appendix1.* for details) at weeks 0, 2, 4, 6, 8 of treatment and week 12 of the follow-up period.

### Primary outcome

Difference in CDRS-R scores at weeks 0 and 8 of treatment.

### Secondary outcomes

①HAMD-17 clinical score reduction rate: Pre-treatment HAMD-17 score-HAMD-17 score after 8 weeks of treatment/Pre-treatment HAMD-17 scores.

②HAMD-17clinical remission rate: proportion of HAMD-17 scores <7 after 8 weeks of treatment.

③Difference in CDRS-R scores at weeks 0, 2, 4, 6, 8, 12.

④Difference in HAMD-17, HAMD-14, PSQI, and C-SSRS scores at weeks 0, 2, 4, 6, 8, 12.

### Feasibility outcomes

①Proportion of participants who completed the required 8-week acupuncture treatment. If≥80% participants completed, proceed; if 50%-80% participants completed, modify protocol; if <50% participants completed, do not proceed.

②Rate of drop-out during treatment. If rate of drop-out ≤ 20%, proceed; if rate of drop-out is 20%-50%, modify protocol; if rate of drop-out>50%, do not proceed.

③Response rate. Response rate of participants to the collected indicators will be evaluated at pre-therapy, post-therapy, and 4 weeks after treatment.

④Blinding test. All the participants will be tested for the success of blinding after the final treatment session. They will be asked to judge whether they have received real acupuncture or sham acupuncture, and we will use the Bang’s blinding indices to assess the adequacy of blinding (23).

⑤Satisfaction. Participants’ satisfaction with the intervention will be ranked on a 5-point scale, each point is very satisfied, satisfied, neutral, dissatisfied, and very dissatisfied.

### Security outcomes

The outcomes of safety evaluation will be recorded, including basic vital signs (blood pressure, respiration, pulse, etc.) before and after treatment, and records of adverse events (AEs) during treatment and follow-up.

Classification of AEs: Serious Adverse Events (SAEs) are adverse medical events such as death, life-threatening, permanent or severe disability or loss of function, subjects requiring hospitalization or prolonged hospitalization, and congenital anomalies or birth defects that occur in the course of a clinical trial. Significant adverse events are defined as any occurrence of AEs other than SAEs that result in the use of targeted medical measures (e.g., cessation of needling, symptomatic treatment) and significant abnormalities in hematology or other laboratory tests. An unintended adverse event (UAE) is an AE that is of a different nature, severity, or frequency than the expected risk described in a previous protocol or other relevant information (e.g., investigator’s brochure, drug insert).

Handling of AEs: When a participant has trial-related AEs, including clinically significant abnormalities in laboratory tests, the participant should be assured of appropriate medical treatment, such as stopping the needling, decreasing the frequency, or symptomatic treatment. At the same time, to avoid more serious consequences of the clinical trial, the researcher should decide whether to terminate or suspend the clinical trial in order to protect the rights and safety of the participants.

Recording of AEs: Medical documents related to AE, including request forms for laboratory tests and report forms for test results, should be kept in the original files, and the original records should be kept true, accurate, and complete. All records should be signed and dated by the researcher. The content of AE record includes the name of the AE described in medical terminology, clinical signs/symptoms, start and end time of occurrence, severity, measures taken, and the outcome, etc., and the relevance to the trial protocol should be judged. Measures taken mainly include: no measures taken, adjustment of trial parameters, cessation of needling, combination of medications, hospitalization, or prolongation of hospitalization, etc. AE outcomes are mainly cured, improved, not cured, worsened, died, lost to visit, or unknown.

### Statistical considerations

#### Sample size

This trial is a pilot trial aim to preliminarily explore the possibility of acupuncture in the treatment of ADD, and to evaluate the feasibility of conducting further large-scale clinical trials to verify the efficacy of acupuncture. Moreover, the results of this trial will facilitate the calculation of an appropriate sample size for further randomized clinical trials. The minimum sample size for exploratory trials is 20 to 30 per group according to Provisions for Drug Registration in China. It is generally accepted that at least 30 participants are required for a pilot study (24). According to an estimate of the number of patients expected to participate in the trial and the minimum number of patients required to assess the practical purpose of the trial., we choose the maximum of 30 participants, and the sample size of 60 participants is determined.

### Statistical analysis

The primary and secondary outcomes of this study will be analyzed using the modified intention to score principle. The other statistical analyses including feasibility outcomes and security outcomes. And the safety analysis will include all patients who have received at least one time of intervention.

Analyses of the primary outcomes will be conducted using linear mixed-effects models (MMRM) and generalized linear mixed models (GLMM) in repeated-measures design, which incorporate the clinical effect rates at weeks 0, 2, 4, 6, 8, and the follow-up period as response variables, as well as baseline values, time with treatment factors, time, and treatment factors as fixed-effects variables for the analyses. This study will assume that missing indicators are randomized, and due to the ability of the MMRM to cope with randomly missing data, this study will not conduct data filling for the primary outcome.

Reliability of our data will be analyzed using Cronbach’s Alpha, which is a commonly used indicator for assessing the internal consistency of a scale (25), and data assessed through the scale are considered reliable only when it is >0.7.

Three sensitivity analyses will be conducted in this study for the primary outcomes. First, the study will exclude missing data and analyzes the complete case dataset. Second, a multiple filling method will be used to fill in the outcomes before conducting the evaluation. Third, the primary outcome will be analyzed based on the set of compliant scenarios. In addition, the study will also conduct subgroup analyses of the primary outcome, which will include gender, age, hospital district, BMI, duration of disease, comorbidities, and medication use. For the analysis of secondary outcome, repeated measures information will also be based on the MMRM method; for other quantitative data outcomes, this study will use the Mann-Whitney U test or t-test, depending on whether the data are normally distributed or not.

Statistical descriptions will be carried out for the adverse reaction indicators, and the method of chi-square test or Fisher’s exact probability will be used to compare the differences between groups.

The study will use the R programming language to carry out the statistical analysis. The test level α is 0.05, and all secondary outcome analyses as well as subgroup analyses will not be adjusted for multiple comparisons α, so the results of the evaluation of secondary outcomes and subgroup analyses will be considered exploratory.

### Data management and quality control

All enrolled patients will be measured on the scale by professional researchers on the day of enrollment. The researchers are postgraduate students of psychiatry related majors in universities and psychologists in tertiary hospitals. All researchers will receive systematic training in scale measurement to ensure the standardization of scale measurement and data collection:

1. The clinical scales used in this trial have been widely used nationally and internationally and have good reliability and validity.
2. The researcher will explain the scales and questionnaires using a uniform guideline, and patients will complete the questionnaires and scales under the guidance of the researcher after fully understanding the individual entries.
3. This trial will ensure that all patients will be administered the questionnaire in a quiet indoor environment and the questionnaire will be collected on the spot upon completion to improve data integrity.
4. Questionnaires that meet the following eligibility criteria will be included as valid questionnaires: The age input for the questionnaire is consistent with the age range of the adolescents; The questionnaire is complete with no blanks; The questionnaire is answered carefully and there are no obvious contradictions or patterns in the answers (no more than 10 consecutive questions in one or more scales with the same answer selected).
5. The same brand and model of the therapeutic instrument will be used for treatment to standardize measurement standards and control errors.
6. Statistical data will be entered and checked for accuracy by two persons, and all retrieved data information will be coded, kept, and organized by a specialist.

## Ethics and dissemination

Participants under the age of 18 will be enrolled in this trial, and written consent must be provided by a guardian in addition to the participant’s own informed consent (see *Appendix2.* for details). The trial protocol has been approved by the Ethics Committee of the Affiliated Traditional Chinese Medicine Hospital, Guangzhou Medical University (approval number 2024NK73). The principle of informed consent is strictly enforced in research, and informed consent documents will be used to explain to participants in concise terms the dedication and benefits of participating in research and how their rights and interests will be protected prior to their participation in the trial. The researchers will be responsible for checking that informed consent has been obtained from each participant or their legal representative and that the informed consent document is properly signed and dated prior to any step in the trial protocol and prior to administration of the trial medication. This trial will be conducted in compliance with the ethical principles declared in the latest version of the Declaration of Helsinki, the corresponding guidelines of the Code of Clinical Trial Practice, as well as the laws and regulations of China, to provide protection for the participants. The results of this trial will be made publicly available on the registration platform. There will be no use of any professional writers for this trial. Future authorship of trial publications will be based on the authors’ contributions.

## Discussion

Acupuncture has been documented to treat mood disorders since ancient times. Recent studies have shown that acupuncture has the advantages of low cost and fewer side effects compared to pharmacotherapy (26), so acupuncture is considered a promising non-pharmacological treatment to reduce depressive symptoms and can be used as an alternative or complementary treatment to pharmacotherapy to improve outcomes (27). And research on its mechanism of effect is gradually increasing.

It has been suggested that the antidepressant effects of acupuncture can be explained by the regulation of neurotransmitters, gut flora (28), neuroplasticity (29), inflammation (30), and other molecular mechanisms. Results from animal experiments suggest a multitarget antidepressant effect of acupuncture, which may be related to amino acid metabolism and inflammatory pathways (31). In addition, similar to antidepressant medications, acupuncture is capable of affecting the neurotransmitter levels of serotonin and noradrenaline (32). And for adolescents, fluctuations in neurotransmitter systems, such as GABA, NMD, and dopamine have profound impact on neural signaling in regions pertaining to emotion regulation. GABAergic and glutamatergic systems in the PFC and cingulate cortices have a direct impact on the excitability and plasticity in regions subserving emotion regulation (33). Thus, acupuncture may be able to treat ADD by producing fluctuations in the neurotransmitter system to achieve a mood-modulating effect. There’s also the fact that synapses in the adolescent brain are highly dynamic, new synapses are formed and others eliminated at higher rates than seen in adults (34) (35). So, adolescents may be more sensitive to the modulating effects of the neurotransmitter system produced by acupuncture. However, there is a lack of clinical evidence about whether acupuncture can treat adolescent depression at present.

Traditional Chinese medicine (TCM) believes that brain is the house of original spirit, which means that the brain can govern the body’s conscious thought and mental activity, also play a key role in regulating emotional responses (36). Therefore, acupuncture on meridians and points related to the brain can achieve the effect of regulating and awakening the mind as well as regulating emotions. So, we choose acupoints in the head, GV20 and GV29, to achieve the effect of inducing resuscitation and improving mood. “Heart stores spirit”, means that the heart has the function of governing life activities, regulating feelings thinking cognition, and dominating emotional activities (37). HT7, the gateway for the entry and exit of the mind and spirit, can nourish heart and tranquilize the mind. In general, TCM considers the pathogenesis of depression to be stagnation of qi, imbalance of yin, yang, qi and blood in the zang-fu organs (38). Therefore, we choose CV17, PC6, LR3, and SP6 these acupoints to regulate qi and blood, restore the balance of yin and yang in the organism and achieve the effect of improving depression.

There are certain limitations that exist with this trial. Firstly, this trial will only involve a 4-week post-treatment follow-up and will not involve evaluation of long-term outcomes and recurrence. Secondly, this trial will be evaluated using subjective scales, and the participants included are relatively young, which may bias the results due to comprehension error. Thirdly, this trial will be confined to a single-center trial involving 60 patients, potentially introducing selection bias. Nevertheless, for future trials, we aim to recruit patients from multiple centers in a significantly larger sample size to mitigate these limitations
