# Supplementary material for: Acupuncture for Adolescent Depression Disorder: protocol for a randomized controlled trial
Source: Front Psychiatry. 2025 Jul 14;16:1597093. doi: 10.3389/fpsyt.2025.1597093 (PMC12302751; doi:10.3389/fpsyt.2025.1597093)
Supplement: Supplementary file 1 [file DataSheet1.pdf]

Time（week） / content	Baseline	Treatment period				Follow-up
	**0**	**2**	**4**	**6**	**8**	**12**
Patient Consent Form	√					
Enrollment Screening Form	√					
Demographic information	√					
History of present illness	√					
Family history	√					
Past history	√					
Personal history	√					
CDRS-R	√	√	√	√	√	√
HAMD-17	√	√	√	√	√	√
HAMA-14	√	√	√	√	√	√
PSQI	√	√	√	√	√	√
C-SSRS	√	√	√	√	√	√
Safety evaluation	√				√	
Random form	√					
Drug combination	√					
Adverse Event record form	√					
Serious Adverse Event record form	√					
Table of trial completion and early termination	√					
CRF Audit Statement	√					
