# Supplementary material for: Acupuncture for Adolescent Depression Disorder: protocol for a randomized controlled trial
Source: Front Psychiatry. 2025 Jul 14;16:1597093. doi: 10.3389/fpsyt.2025.1597093 (PMC12302751; doi:10.3389/fpsyt.2025.1597093)
Supplement: Supplementary file 2 [file DataSheet2.pdf]

Children's Depression Rating Scale-revised（CDRS-R)

Instructions: The items 4, 5, and 16 are rated from 1 to 5 and all others are rated from 1 to 7 with higher scores indicating increased pathology.			
**Item**	**Score**	**Item**	**Score**
1 Impaired schoolwork	1 □ 2 □ 3 □ 4 □ 5 □ 6 □ 7 □	2 Difficulty having fun	1 □ 2 □ 3 □ 4 □ 5 □ 6 □ 7 □
3 Social withdrawal	1 □ 2 □ 3 □ 4 □ 5 □ 6 □ 7 □	4 Sleep disturbance	1 □ 2 □ 3 □ 4 □ 5 □
5 Appetite disturbance	1 □ 2 ☐ 3 □ 4 □ 5 □	6 Excessive fatigue	1 □ 2 □ 3 □ 4 □ 5 □ 6 □ 7 □
7 Physical symptoms	1 □ 2 □ 3 □ 4 □ 5 □ 6 □ 7 □	8 Irritability	1 □ 2 □ 3 □ 4 □ 5 □ 6 □ 7 □
9 Excessive guilt	1 □ 2 □ 3 □ 4 □ 5 □ 6 □ 7 □	10 Low self-esteem	1 □ 2 □ 3 □ 4 □ 5 □ 6 □ 7 □
11 Depressed feelings	1 □ 2 □ 3 □ 4 □ 5 □ 6 □ 7 □	12 Morbid ideation	1 □ 2 □ 3 □ 4 □ 5 □ 6 □ 7 □
13 Suicidal ideation	1 □ 2 □ 3 □ 4 □ 5 □ 6 □ 7 □	13 Excessive weeping	1 □ 2 □ 3 □ 4 □ 5 □ 6 □ 7 □
15 Depressed facial expression	1 □ 2 □ 3 □ 4 □ 5 □ 6 □ 7 □	16 Listless speech	1 □ 2 □ 3 □ 4 □ 5 □
17 Hypoactivity	1 □ 2 □ 3 □ 4 □ 5 □ 6 □ 7 □		
overall score：			

17-item Hamilton Depression Scale (HAMD-17)

**0: no symptoms 1: mild 2: moderate 3: severe 4: very severe**			
**Item**	**Score**	**Item**	**Score**
1．Depression mood	0□ 1□ 2□ 3□ 4□	2．Guilt feelings	0 □ 1 □ 2 □ 3 □
3．Suicide	0□ 1□ 2□ 3□ 4□	4．Insomnia-Early	0 □ 1 □ 2 □
5．Insomnia-Middle	0 □ 1 □ 2 □	6．Insomnia-Late	0 □ 1 □ 2 □
7．Work and activities	0□ 1□ 2□ 3□ 4□	8．Psychomotor retardation	0□ 1□ 2□ 3□ 4□
9．Psychomotor agitation	0□ 1□ 2□ 3□ 4□	10. Anxiety-psychic	0□ 1□ 2□ 3□ 4□
11．Anxiety-somatic	0□ 1□ 2□ 3□ 4□	12. Somatic symptoms-gastrointestinal	0 □ 1 □ 2 □
13．General somatic symptoms	0 □ 1 □ 2 □	14．Genital Symptoms	0 □ 1 □ 2 □
15．Hypochondriasis	0□ 1□ 2□ 3□ 4□	16．Weight loss	0 □ 1 □ 2 □
17．Insight	0 □ 1 □ 2 □		

overall score：

Very severe depression (26 ≤ total score) □ Severe depression 20 ≤ total score <26) □

Moderate depression (14 ≤ total score <20) □ Mild depression (8 ≤ total score <14) □

No depressive symptoms (total score <8) □

14-item Hamilton Anxiety Scale (HAMA-14)

**0: no symptoms 1: mild 2: moderate 3: severe 4: very severe**			
**Item**	**Score**	**Item**	**Score**
1. Anxious mood	0□ 1□ 2□ 3□ 4□	2. Tension	0□ 1□ 2□ 3□ 4□
3. Fears	0□ 1□ 2□ 3□ 4□	4. Insomnia	0□ 1□ 2□ 3□ 4□
5. Cognitive	0□ 1□ 2□ 3□ 4□	6. Depression mood	0□ 1□ 2□ 3□ 4□
7. Somatic anxiety: muscular	0□ 1□ 2□ 3□ 4□	8. Somatic anxiety: sensory	0□ 1□ 2□ 3□ 4□
9. Cardiovascular-symptoms	0□ 1□ 2□ 3□ 4□	10. Respiratory symptoms	0□ 1□ 2□ 3□ 4□
11. Gastro-intestinal symptoms	0□ 1□ 2□ 3□ 4□	12. Genito-urinary symptoms	0□1□2□3□4□
13.Autonomic symptoms	0□ 1□ 2□ 3□ 4□	14. Behavior at interview	0□ 1□ 2□ 3□ 4□
overall score: □ □ Severe anxiety (29<total score)□ Marked anxiety (21<total score≤29)□ Definite anxiety (14<total score≤21)□ Mild or no anxiety (total score≤14)□			

Pittsburgh Sleep Quality Index (PSQI)

Instructions: The following questions relate to your usual sleep habits during the past month only. Your answers should indicate the most accurate reply for the majority of days and nights in the past month. Please answer all questions. During the past month,

1. When have you usually gone to bed? _______________
2. How long (in minutes) has it taken you to fall asleep each night? _______________
3. When have you usually gotten up in the morning? _______________
4. How many hours of actual sleep do you get at night? (This may be different than the number of hours you spend in bed) _______________

5. During the past month, how often have you had trouble sleeping because you…	Not during the past month (0)	Less than once a week (1)	Once or twice a week (2)	Three or more times a week (3)
a. Cannot get to sleep within 30 minutes				
b. Wake up in the middle of the night or early morning				
c. Have to get up to use the bathroom				
d. Cannot breathe comfortably				
e. Cough or snore loudly				
f. Feel too cold				
g. Feel too hot				
h. Have bad dreams				
i. Have pain				
j. Other reason(s), please describe, including how often you have had trouble sleeping because of this reason(s):				
6. During the past month, how often have you taken medicine (prescribed or "over the counter") to help you sleep?				
7. During the past month, how often have you had trouble staying awake while driving, eating meals, or engaging in social activity?				
8. During the past month, how much of a problem has it been for you to keep up enthusiasm to get things done?				
	Very good (0)	Fairly good (1)	Fairly bad (2)	Very bad (3)
9. During the past month, how would you rate your sleep quality overall?				

Component 1 #9 Score ....C1_______

Component 2 #2 Score (≤15min=0; 16-30 min=1; 31-60 min=2, >60 min=3) + #5a Score
(if sum is equal 0=0; 1-2=1; 3-4=2; 5-6=3) ....C2_______

Component 3 #4 Score (>7=0; 6-7=1; 5-6=2; <5=3) ....C3_______

Component 4 (total # of hours asleep)/(total # of hours in bed) x 100
>85%=0, 75%-84%=1, 65%-74%=2, <65%=3 .... C4_______

Component 5 Sum of Scores #5b to #5j (0=0; 1-9=1; 10-18=2; 19-27=3) .... C5_______

Component 6 #6 Score .... C6______

Component 7 #7 Score + #8 Score (0=0; 1-2=1; 3-4=2; 5-6=3) .... C7_______

Add the seven component scores together ________ Global PSQI Score ________

Columbia-Suicide Severity Rating Scale (C-SSRS)

SUICIDAL IDEATION

Ask questions 1 and 2. If both are negative, proceed to "Suicidal Behavior" section. If the answer to question 2 is "yes", ask questions 3, 4 and 5. If the answer to question 1 and/or 2 is "yes", complete "Intensity of Ideation" section below.	**Since Last Visit**
**1. Wish to be Dead**<br>Subject endorses thoughts about a wish to be dead or not alive anymore, or wish to fall asleep and not wake up.<br>***Have you wished you were dead or wished you could go to sleep and not wake up?***<br><br>If yes, describe:	**Yes** ☐ **No** ☐
**2. Non-Specific Active Suicidal Thoughts**<br>General non-specific thoughts of wanting to end one's life/commit suicide (e.g., *"I've thought about killing myself"*) without thoughts of ways to kill oneself/associated methods, intent, or plan during the assessment period.<br>***Have you actually had any thoughts of killing yourself?***<br><br>If yes, describe:	**Yes** ☐ **No** ☐
**3. Active Suicidal Ideation with Any Methods (Not Plan) without Intent to Act**<br>Subject endorses thoughts of suicide and has thought of at least one method during the assessment period. This is different than a specific plan with time, place or method details worked out (e.g., thought of method to kill self but not a specific plan). Includes person who would say, *"I thought about taking an overdose but I never made a specific plan as to when, where or how I would actually do it…..and I would never go through with it"*.<br>***Have you been thinking about how you might do this?***<br><br>If yes, describe:	**Yes** ☐ **No** ☐
**4. Active Suicidal Ideation with Some Intent to Act, without Specific Plan**<br>Active suicidal thoughts of killing oneself and subject reports having <u>some intent to act on such thoughts</u>, as opposed to *"I have the thoughts but I definitely will not do anything about them"*.<br>***Have you had these thoughts and had some intention of acting on them?***<br><br>If yes, describe:	**Yes** ☐ **No** ☐
**5. Active Suicidal Ideation with Specific Plan and Intent**<br>Thoughts of killing oneself with details of plan fully or partially worked out and subject has some intent to carry it out.<br>***Have you started to work out or worked out the details of how to kill yourself? Do you intend to carry out this plan?***<br><br>If yes, describe:	**Yes** ☐ **No** ☐

INTENSITY OF IDEATION

The following features should be rated with respect to the most severe type of ideation (i.e.,1-5 from above, with 1 being the least severe and 5 being the most severe).<br>***Most Severe Ideation:*** ________ ____________________<br>***Type # (1-5)*** ***Description of Ideation***	Most Severe
**Frequency**<br>***How many times have you had these thoughts?***<br>(1) Less than once a week (2) Once a week (3) 2-5 times in week (4) Daily or almost daily (5) Many times each day	____
**Duration**<br>***When you have the thoughts how long do they last?***<br>(1) Fleeting - few seconds or minutes<br>(2) Less than 1 hour/some of the time<br>(3) 1-4 hours/a lot of time<br>(4) 4-8 hours/most of day<br>(5) More than 8 hours/persistent or continuous	____
**Controllability**<br>***Could/can you stop thinking about killing yourself or wanting to die if you want to?***<br>(1) Easily able to control thoughts<br>(2) Can control thoughts with little difficulty<br>(3) Can control thoughts with some difficulty<br>(4) Can control thoughts with a lot of difficulty<br>(5) Unable to control thoughts<br>(0) Does not attempt to control thoughts	____
**Deterrents**<br>***Are there things - anyone or anything (e.g., family, religion, pain of death) - that stopped you from wanting to die or acting on thoughts of committing suicide?***<br>(1) Deterrents definitely stopped you from attempting suicide<br>(2) Deterrents probably stopped you<br>(3) Uncertain that deterrents stopped you<br>(4) Deterrents most likely did not stop you<br>(5) Deterrents definitely did not stop you<br>(0) Does not apply	____
**Reasons for Ideation**<br>***What sort of reasons did you have for thinking about wanting to die or killing yourself? Was it to end the pain or stop the way you were feeling (in other words you couldn't go on living with this pain or how you were feeling) or was it to get attention, revenge or a reaction from others? Or both?***<br>(1) Completely to get attention, revenge or a reaction from others<br>(2) Mostly to get attention, revenge or a reaction from others<br>(3) Equally to get attention, revenge or a reaction from others and to end/stop the pain<br>(4) Mostly to end or stop the pain (you couldn't go on living with the pain or how you were feeling)<br>(5) Completely to end or stop the pain (you couldn't go on living with the pain or how you were feeling)<br>(0) Does not apply	____

© 2008 Research Foundation for Mental Hygiene, Inc.

SUICIDAL BEHAVIOR

(Check all that apply, so long as these are separate events; must ask about all types)

Since Last Visit

Actual Attempt:

A potentially self-injurious act committed with at least some wish to die, *as a result of act*. Behavior was in part thought of as method to kill oneself. Intent does not have to be 100%. If there is ***any*** intent/desire to die associated with the act, then it can be considered an actual suicide attempt. ***There does not have to be any injury or harm***, just the potential for injury or harm. If person pulls trigger while gun is in mouth but gun is broken so no injury results, this is considered an attempt.

Inferring Intent: Even if an individual denies intent/wish to die, it may be inferred clinically from the behavior or circumstances. For example, a highly lethal act that is clearly not an accident so no other intent but suicide can be inferred (e.g., gunshot to head, jumping from window of a high floor/story). Also, if someone denies intent to die, but they thought that what they did could be lethal, intent may be inferred.

Have you made a suicide attempt?
Have you done anything to harm yourself?
Have you done anything dangerous where you could have died?

> ***What did you do?***
> ***Did you______as a way to end your life?***
> ***Did you want to die (even a little) when you_____?***
> ***Were you trying to end your life when you_____?***
> ***Or Did you think it was possible you could have died from_____?***

Or did you do it purely for other reasons / without ANY intention of killing yourself (like to relieve stress, feel better, get sympathy, or get something else to happen)? (Self-Injurious Behavior without suicidal intent)

If yes, describe:

Yes ☐ **No** ☐

Total # of Attempts ______

Has subject engaged in Non-Suicidal Self-Injurious Behavior?

Yes ☐ **No** ☐

Interrupted Attempt:

When the person is interrupted (by an outside circumstance) from starting the potentially self-injurious act *(if not for that, actual attempt would have occurred)*.

Overdose: Person has pills in hand but is stopped from ingesting. Once they ingest any pills, this becomes an attempt rather than an interrupted attempt. Shooting: Person has gun pointed toward self, gun is taken away by someone else, or is somehow prevented from pulling trigger. Once they pull the trigger, even if the gun fails to fire, it is an attempt. Jumping: Person is poised to jump, is grabbed and taken down from ledge. Hanging: Person has noose around neck but has not yet started to hang - is stopped from doing so.

Has there been a time when you started to do something to end your life but someone or something stopped you before you actually did anything?

If yes, describe:

Yes ☐ **No** ☐

Total # of interrupted ______

Aborted Attempt:

When person begins to take steps toward making a suicide attempt, but stops themselves before they actually have engaged in any self-destructive behavior. Examples are similar to interrupted attempts, except that the individual stops him/herself, instead of being stopped by something else.

Has there been a time when you started to do something to try to end your life but you stopped yourself before you actually did anything?

If yes, describe:

Yes ☐ **No** ☐

Total # of aborted ______

Preparatory Acts or Behavior:

Acts or preparation towards imminently making a suicide attempt. This can include anything beyond a verbalization or thought, such as assembling a specific method (e.g., buying pills, purchasing a gun) or preparing for one's death by suicide (e.g., giving things away, writing a suicide note).

Have you taken any steps towards making a suicide attempt or preparing to kill yourself (such as collecting pills, getting a gun, giving valuables away or writing a suicide note)?

If yes, describe:

Yes ☐ **No** ☐

Suicidal Behavior:

Suicidal behavior was present during the assessment period?

Yes ☐ **No** ☐

Suicide:

Yes ☐ **No** ☐

Answer for Actual Attempts Only

Most Lethal Attempt Date:

Actual Lethality/Medical Damage:

0. No physical damage or very minor physical damage (e.g., surface scratches).
1. Minor physical damage (e.g., lethargic speech; first-degree burns; mild bleeding; sprains).
2. Moderate physical damage; medical attention needed (e.g., conscious but sleepy, somewhat responsive; second-degree burns; bleeding of major vessel).
3. Moderately severe physical damage; *medical* hospitalization and likely intensive care required (e.g., comatose with reflexes intact; third-degree burns less than 20% of body; extensive blood loss but can recover; major fractures).
4. Severe physical damage; *medical* hospitalization with intensive care required (e.g., comatose without reflexes; third-degree burns over 20% of body; extensive blood loss with unstable vital signs; major damage to a vital area).
5. Death

Enter Code ______

Potential Lethality: Only Answer if Actual Lethality=0

Likely lethality of actual attempt if no medical damage (the following examples, while having no actual medical damage, had potential for very serious lethality: put gun in mouth and pulled the trigger but gun fails to fire so no medical damage; laying on train tracks with oncoming train but pulled away before run over).

0 = Behavior not likely to result in injury
1 = Behavior likely to result in injury but not likely to cause death
2 = Behavior likely to result in death despite available medical care

Enter Code ______
